# Supragingival mycobiome of HIV-exposed-but-uninfected children reflects a stronger correlation with caries-free-associated taxa compared to HIV-infected or uninfected children

**DOI:** 10.1128/spectrum.01491-23

**Published:** 2023-10-24

**Authors:** Lauren M. O'Connell, Allison E. Mann, Esosa Osagie, Paul Akhigbe, Thomas Blouin, Ashlyn Soule, Ozoemene Obuekwe, Augustine Omoigberale, Robert A. Burne, Modupe O. Coker, Vincent P. Richards

**Affiliations:** 1 Department of Biological Sciences, Clemson University, Clemson, South Carolina, USA; 2 Institute of Human Virology Nigeria, Abuja, Nigeria; 3 Department of Oral and Maxillofacial Surgery, University of Benin Teaching Hospital, Benin, Edo State, Nigeria; 4 Department of Child Health, University of Benin Teaching Hospital, Benin, Edo State, Nigeria; 5 Department of Oral Biology, College of Dentistry, University of Florida, Gainesville, Florida, USA; 6 Department of Oral Biology, School of Dental Medicine, Rutgers University, Newark, New Jersey, USA; State Key Laboratory of Food Science and Technology, Nanchang, Jiangxi, China

**Keywords:** oral mycobiome, HIV, caries, ITS

## Abstract

Highly active antiretroviral treatment (HAART) has greatly reduced opportunistic infections in HIV-infected individuals. However, even with the use of HAART, HIV-infected individuals are still at an increased risk of oral diseases, including dental caries. Dental caries development is associated with microbial community shifts leading to community dysbiosis. HIV infection can significantly alter the bacteriome and mycobiome composition in the oral cavity; however, these impacts have not been assessed for caries initiation and progression. The aim of this study was to characterize the mycobiome for supragingival plaque samples from HIV-infected (HI), HIV-exposed-but-uninfected (HEU), and HIV-unexposed-and-uninfected (HUU) children with and without caries. To accomplish this, ITS1 amplicons of 127 samples were sequenced. We found that HIV infection and exposure resulted in changes to the supragingival plaque mycobiome for both health and caries. Overall, a reduction in community diversity as caries progressed was observed, with HI children having the lowest diversity and HEU children the highest. *Candida albicans* was the most abundant species identified, with 177 different amplicon sequence variants (ASVs). Two *C. albicans* ASVs dominated the data (53.0% and 38.5% of all taxonomic assignments). The more frequent ASV dominated HI and HUU communities, whereas the second dominated HEU communities. HEU children also had the lowest abundance of *C. albicans* and the highest number of health-associated taxa (taxa statistically associated with caries-free teeth).

**IMPORTANCE:**

Globally, caries is among the most frequent chronic childhood disease, and the fungal component of the microbial community responsible is poorly studied despite evidence that fungi contribute to increased acid production exacerbating enamel demineralization. HIV infection is another global health crisis. Perinatal HIV exposure with infection are caries risk factors; however, the caries experience in the context of perinatal HIV exposure without infection is less clear. Using high-throughput amplicon sequencing, we find taxonomic differences that become pronounced during late-stage caries. Notably, we show a stronger correlation with health-associated taxa for HIV-exposed-but-uninfected children when compared to unexposed and uninfected children. This aligns with a lower incidence of caries in primary teeth at age 6 or less for exposed yet uninfected children. Ultimately, these findings could contribute to improved risk assessment, intervention, and prevention strategies such as biofilm disruption and the informed design of pro-, pre-, and synbiotic oral therapies.

## INTRODUCTION

Early childhood caries (ECC) is one of the most prevalent diseases worldwide (1 – 3). Despite its preventability, ECC remains a public health problem that can have serious lasting effects including disruptions to development, malnutrition, and decreased quality of life (3, 4). Untreated dental caries can also lead to serious financial, health, and social burdens (4). Caries is a complex, polymicrobial, and sugar-driven disease associated with a dysbiotic shift of the supragingival plaque microbiome toward one that favors acidogenic and aciduric taxa (5 – 7). While earlier studies have focused on the oral bacteriome associated with dental caries, more recently, the importance of the oral fungal microbiome in caries initiation and progression has been examined (8 – 11).

*Candida albicans* is one of the most common fungal pathogens in human disease (12, 13). It is a human commensal polymorphic fungus that under certain conditions can be pathogenic and cause severe infections (12). High levels of *C. albicans* have been isolated from carious lesions in conjunction with *Streptococcus mutans* (14 – 16). Synergistic interactions between the oral streptococci and *C. albicans* have been identified and can lead to a highly acidogenic biofilm, enhanced sugar metabolism*,* and increased severity of caries (17 – 20).

HIV infection is a major global public health crisis with 36 million adults and 1.7 million children living with HIV (21). Within the last decade, increased availability of antiretrovirals (ARTs) has decreased rates of mother-to-child HIV transmission (PMTCT). This has resulted in declining rates of infants infected with HIV but has increased the number of perinatally HIV-exposed-but-uninfected (HEU) children (4, 22, 23). Several studies throughout this time have found evidence that HEU infants are at a higher risk for increased mortality, increased susceptibility to infections, and increased risk of immunological impairments when compared to unexposed children (22, 24). In addition to this, multiple recent studies have reported an increased prevalence of dental caries in HIV-infected (HI) children when compared to HIV-unexposed-and-uninfected (HUU) children (4, 25 – 27). Our work with a large cohort of children in Nigeria is one of the few studies to examine the impacts of HIV exposure on dental caries risk (despite the increasing population of HEU children). Specifically, our cohort consisted of 568 children (HI = 189, HEU = 189, and HUU = 190), and for permanent and primary teeth in children older than 6 years, HI children had the highest incidence of caries followed by HEU and then HUU. However, for primary teeth in children 6 years or younger, caries prevalence was lowest for HEU (HEU < HUU < HI). These findings highlight the need for a better understanding of the impact of HIV exposure on the oral microbiome and its relation to caries.

Currently, no study has examined the fungal mycobiome in HI, HEU, and HUU children with ECC, despite the growing evidence that it likely plays a role in caries initiation, progression, and severity (9, 28). This study used amplicon sequencing of the fungal ITS1 region to characterize the fungal community in supragingival plaque samples from HI, HEU, and HUU children through six progressive stages of caries. Results revealed that *C. albicans* was the most abundant fungal species identified in all samples. The dominant *C. albicans* amplicon sequence variant (ASV) in HI and HUU children differed from the dominant *C. albicans* ASV in HEU children. In addition, HEU children had the highest diversity in their fungal supragingival plaque community and showed a strong correlation with a health-associated mycobiome, harboring more taxa found to be prevalent in healthy teeth when compared to HUU and HI children. These results suggest that HEU children may be less susceptible to caries. Further research is needed to elucidate the mechanisms driving these community differences.

## RESULTS

### Sample demographic

Supragingival plaque samples were categorized according to tooth health (healthy tooth, enamel lesion, and dentin lesion—see Methods for a detailed description). Consequently, it was possible to obtain samples with different categorizations from the same child, and 127 samples were sequenced from 92 children. The mean (± sd) age of the children was 6.58 ± 1.92 years. A total of 52% were female and 48% were male. Thirty children (33%) were HUU, 29 (31%) were HEU, and 33 (36%) were HI. Regarding caries, 45 (49%) of the children were caries-free (CF), 8 (9%) had active enamel caries lesions (CAE), and 39 (42%) had active dentin caries lesions (CA). (Table 1; Table S1).

**TABLE 1:** Summary demographics of patient samples

		*n*	%
Sex	Female	48	52
	Male	44	48
Delivery method		*n*	%
	Vaginal	109	86
	C-section	14	11
	Emergency C-section	4	3
HIV infection and exposure status		*n*	%
	HUU	30	33
	HEU	29	31
	HI	33	36
CD4 count ≤ 500 cells/mm^3^		*n*	%
	HUU	2	14
	HEU	0	0
	HI	12	86
CD4 count >500 cells/mm^3^		*n*	%
	HUU	28	36
	HEU	29	37
	HI	21	27
Caries experience		*n*	%
	CF	45	49
	CAE	8	9
	CA	39	42

### Sequencing information

A total of 17,415,324 reads were generated with an average of 136,057 reads per sample. Following sequencing quality control, the mean number of reads per sample was 34,140, with a minimum of 3,901 and a maximum of 135,884.

### Fungal alpha diversity

**Fig 1:**
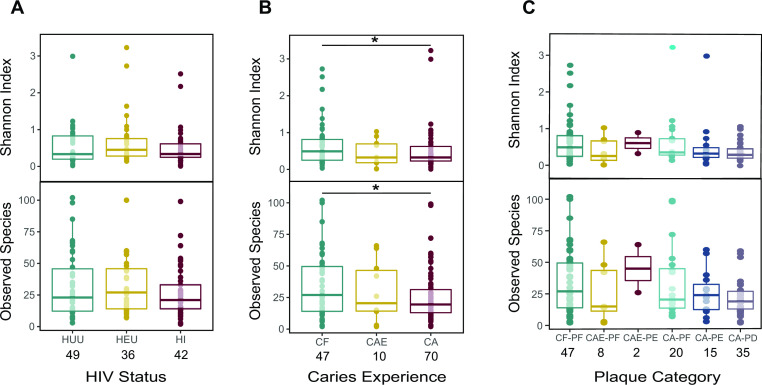
Alpha diversity boxplots (**A**) Top: Shannon diversity and observed species diversity for HIV exposure and infection categories. (**B**) Shannon diversity and observed species diversity for caries experience categories. (**C**) Shannon diversity and observed species diversity for plaque categories. * indicates statistical significance (α < 0.05). Sample sizes for HIV and caries categories are shown under category labels.

**Fig 2:**
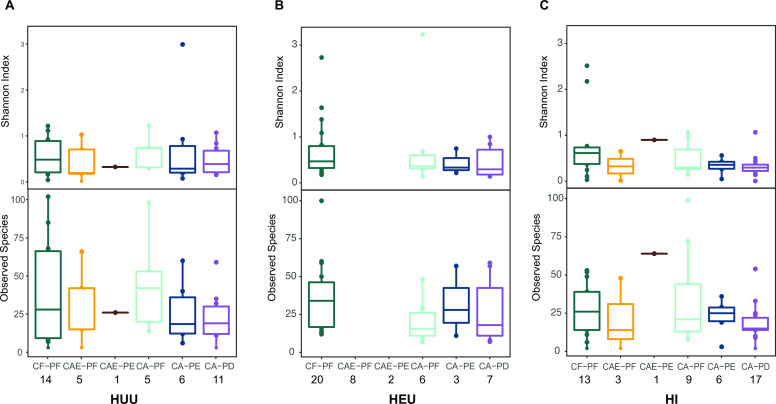
Differences in alpha diversity across the three HIV categories as caries disease progresses. Top: boxplot depicting Shannon diversity and observed species metrics throughout caries progression for the three HIV infection and exposure categories. No significant differences were observed. CAE-PF and CAE-PE boxplots are not present in panel B due to the absence of samples in the two plaque categories for HEU children. Sample sizes for HIV and caries categories are shown under category labels.

Observed species and the Shannon index were used to measure alpha diversity (evenness and richness) in the data. Overall, fungal diversity in all samples was low with a mean Shannon diversity Index of 0.919. Significant differences in alpha diversity were observed between CF and CA samples (*P* = 0.04) (Fig. 1B). The highest alpha diversity observed among the different categories of HIV infection and exposure was in the HEU samples (Shannon entropy = 1.06) while the lowest was observed in HI samples (Shannon entropy = 0.763) (Fig. 1A). The highest alpha diversity observed among the different plaque categories was for CAE-PE (Shannon entropy = 2.11) and the lowest was observed for CA-PD (Shannon entropy = 0.635) (Fig. 1C). We also examined how alpha diversity differed between the different HIV categories as caries disease progressed, and we detected no significant differences (Fig. 2).

### HIV infection and exposure influences beta diversity in CA-PD samples

**Fig 3:**
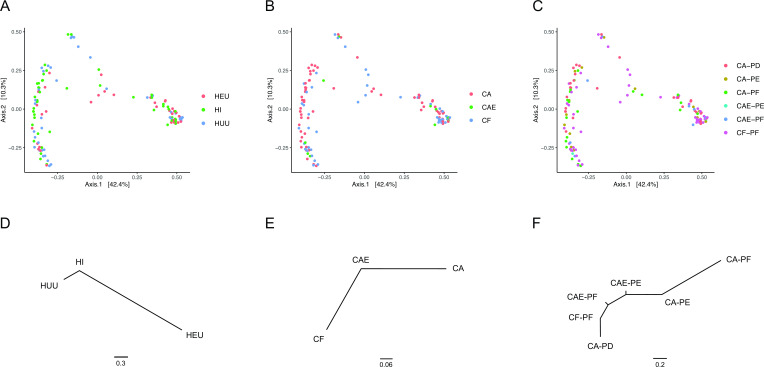
Principal component analysis (PCoA) using log2 transformed Bray–Curtis dissimilarity for the (**A**) three HIV categories, (**B**) tooth status, and (**C**) six plaque categories. For each plot, there is an associated neighbor-joining dendrogram constructed using variance components generated from pairwise PERMANOVA tests (**D, E, and F**). All associated *P*-values were not statistically significant (α < 0.05).

Principle coordinates analysis (PCoA) was performed using Bray–Curtis dissimilarity distances. Permutational multivariate analysis (PERMANOVA) did not show significant dissimilarity among communities based on sex, delivery method, CD4 count, HIV category, caries status, or plaque category. Although significant dissimilarity was not found among these categories, the pairwise PERMANOVA variance components showed that HEU communities were the most dissimilar in their community composition when compared to either HUU or HI communities (Fig. 3A and D). Similarly, CA samples showed the most differentiation when compared to CF samples (Fig. 3B and E), and similarly, CA-PF communities showed the most differentiation to CF-PF communities (Fig. 3C and F).

**Fig 4:**
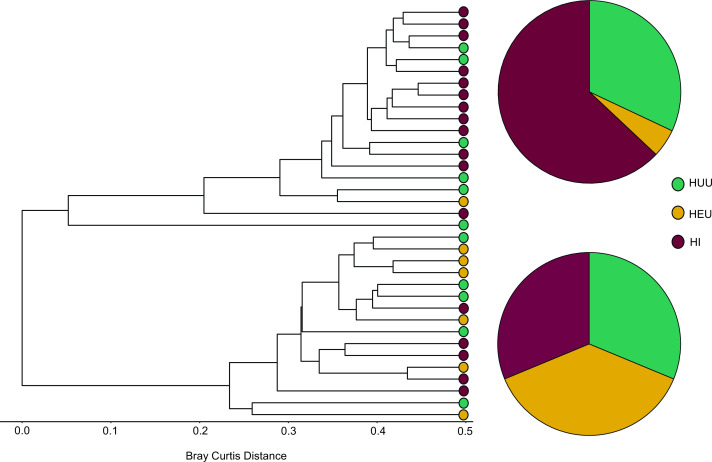
Hierarchical clustering dendrogram of the supragingival plaque mycobiome in HIV infection and exposure categories. Dendrograms are based on Bray–Curtis dissimilarity for CA-PD samples only. The pie charts depict the proportions of the HIV infection and exposure categories for the two major groupings.

Due to the significant differences observed in alpha diversity between CF and CA communities, separate pairwise PERMANOVA were performed comparing HIV categories for CF-only samples and CA-only samples. For CF-only, the results showed significant differentiation when HEU communities were compared to both HI and HUU communities. For CA-only, no significant differentiation was found. Separate PERMANOVA were then performed for CF-PF-only, CA-PF-only, CA-PE-only, and CA-PD-only. Significant dissimilarity in community composition was found in CA-PD-only samples between HEU and HI samples (*P* = 0.04). All other comparisons did not yield any significant differentiation. To further explore the differentiation for CA-PD-only samples, we performed a hierarchical cluster analysis, which recovered two major groupings (clusters) with HEU and HI samples strongly partitioned between the two groups (Fig. 4). In contrast, HUU samples were evenly distributed between the two groups.

### Oral mycobiome taxonomic composition in HIV infection and exposure and caries progression

**Fig 5:**
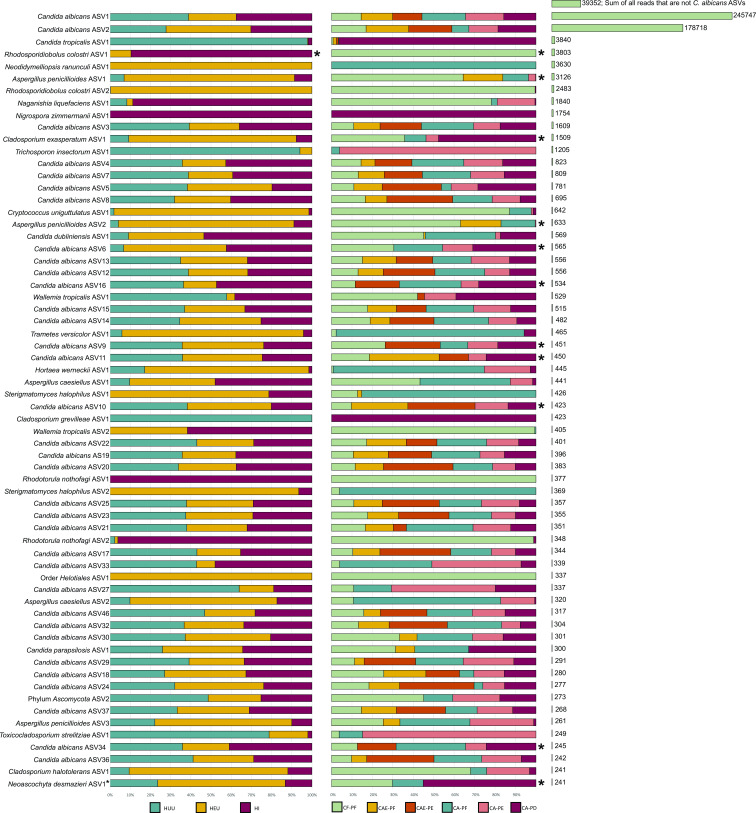
Distribution of the top 64 most abundant taxa in the data set using rarefied taxon counts. (**A**) The left chart shows mean taxon counts for each of the HIV categories. Means are shown as relative proportions. (**B**) The middle chart shows mean taxon counts across the six plaque categories. Means are shown as relative proportions. (**C**) The chart on the right depicts the sum of counts for each taxon; the top bar depicts the sum of all reads that were not assigned to *C. albicans*. An asterisk shows taxa whose frequency was significantly different among the HIV categories (left chart) or plaque category (right chart) (determined using DESeq2 and adjusted for multiple comparisons using Benjamini and Hochberg FDR corrected *P*-values).

Taxonomic assignment of the fungal ITS1 region resulted in a total of 333 ASVs, comprised of 108 species with an average of 28 ASVs and four species per sample. The most abundant species in the data were *C. albicans*, followed by *C. tropicalis, Rhodosporidiobolus colostri, Neodidymelliopsis ranunculi,* and *Aspergillus penicillioides* (Fig. 5). A total of 177 ASVs were assigned to *C. albicans* accounting for 92% of all taxonomic assignments. *C. albicans* ASV1 and *C. albicans* ASV2 were the most abundant ASVs in the data set and accounted for 50.1% and 36.4% of all reads, respectively (Fig. 5C). *C. albicans* ASV1 was the dominant ASV identified in HUU and HI samples whereas *C. albicans* ASV2 was dominant in HEU communities (Fig. 5A). The total number of reads for all *C. albicans* ASVs differed between the three categories. A total of 177,125 *C*. *albicans* reads (93.43% of community) were identified in HI samples, 152,692 reads (94.14% of community) were identified for HUU samples, and 121,031 *C*. *albicans* reads (87.25% of community) were identified for HEU samples (Table S2).

Differential abundance testing of ASVs for HIV exposure and infection categories and plaque categories produced a total of 12 ASVs that were significantly differentially abundant. Overall, there were two significant comparisons between HIV exposure and infection categories and 58 significant comparisons between plaque categories (Table 2; Table S3). In summary, there was one significant comparison between HUU and HEU samples and one significant comparison between HUU and HI samples. A range from zero to eight significant comparisons were observed among the six plaque category comparisons. The highest number of significant comparisons among plaque categories was between CAE-PF and CA-PF (*n* = 8) (Table 2). The only significantly abundant ASV associated with HIV infection or exposure was *R. colostri* ASV1 which was enriched in samples from HEU and HI children. The highest abundance of *R. colostri* was observed in HI samples (Fig. 5A). Four of the 12 differentially abundant ASVs identified between plaque categories (*C. albicans* ASV16, *C. albicans* ASV34, *Cladosporium exasperatum* ASV1, and *Neoascochyta desmazieri* ASV1) were present in highest abundance in children with dentin carious lesions, three were present in highest abundance in healthy mouths (*R. colostri* ASV1 and *A. penicillioides* ASVs 1 and 2)*,* and the remaining four were not strongly correlated with either health or disease (*C. albicans* ASVs 6, 9, 10, and 11) (Fig. 5B). Of the four ASVs enriched in dentin caries, *N. desmazieri* and *C. exasperatum* were most often found in HEU samples and showed similar patterns in their abundance fluctuations throughout the disease with their highest abundance observed in CA-PD samples and their second highest abundance observed in CF-PF samples. The remaining two ASVs, *C. albicans* ASVs 16 and 34, were found in highest abundance in HUU and HI samples. Again, a similar pattern in their abundance fluctuations was observed highest in CA-PF samples, a drop in CA-PE samples followed by a sharp increase in CA-PD samples (Fig. 5). Two of three ASVs (*A. penicillioides* ASVs 1 and 2) enriched in health (CF-PF) were most often observed in HEU samples, while the third, *R. colostri* ASV1, was most often seen in HI samples. *C. albicans* ASV10 was not strongly associated with either health or disease and was found to be most abundant in CAE-PF and CAE-PE samples of HUU and HEU children. This same pattern was also observed for *C. albicans* ASV11. *C. albicans* ASV9 was found in highest abundance in CF-PF and CAE-PE samples of HUU and HEU children. *C. albicans* ASV6 was present in highest abundance in CF-PF samples but was also seen in high abundance in CA-PF and CA-PD samples and was most prevalent in samples from HEU and HI children (Fig. 5).

**TABLE 2:** The number of statistically significant ASVs for each comparison between HIV infection and exposure categories and the different plaque categories determined using DESeq2

Group	HEU	HI			
HUU	1	1			
HEU		0			
					
					

Group	CAE-PF	CAE-PE	CA-PF	CA-PE	CA-PD
CF-PF	7	2	4	3	2
CAE-PF		4	8	4	7
CAE-PE			5	0	0
CA-PF				5	4
CA-PE					3

Lastly, while only 12 ASVs were identified as statistically differentially abundant, there were additional ASVs that showed strong patterns within the data. Five ASVs (*Candida tropicalis* ASV1*, Trichosporon insectorum* ASV1*, Cladosporium grevilleae* ASV1*, C. albicans* ASV27, and *Toxicocladosporium strelitziae* ASV1 were identified in high abundance in HUU samples (Fig. 5A) and were also highly abundant in progressed carious lesions (CA-PE and CA-PD) (Fig. 5B). ASVs that were found to dominate HEU samples included *N. ranunculi* ASV1*, R. colostri* ASV2, *Cryptococcus uniguttulatus* ASV1*, Trametes versicolor* ASV1*, Hortaea werneckii* ASV1*, Sterigmatomyces halophilus* ASVs 1 and 2, *Aspergillus caesiellus* ASV2, and *Cladosporium halotolerans* ASV1. These nine ASVs were highly abundant in samples taken from healthy teeth (CF-PF and CA-PF) (Fig. 5A and B). Lastly, *Wallemia tropicalis* ASV2 and *Rhodotorula nothofagi* ASVs 1 and 2 were abundant in HI samples and strongly correlated with tooth health (CF-PF). In contrast to this, *Nigrospora zimmermanii* ASV1 was highly abundant in HI samples and was strongly correlated with dentin caries (CA-PD). *Naganishia liquefaciens* was highly abundant in HI samples and was strongly correlated with both tooth health (CF-PF) and disease (CA-PE) (Fig. 5A and B).

## DISCUSSION

This study characterized the oral mycobiome throughout caries disease progression in HUU, HEU, and HI children on HAART using amplicon sequencing of the fungal ITS1 region. There are very few studies that have examined the fungal dental plaque mycobiome in relation to caries or HIV infection and exposure, and none have examined both simultaneously (8, 9, 11, 29 – 31). In this study, we identified a total of 108 species and 333 ASVs. This is comparable to the species diversity observed in previous studies for oral fungal communities (8, 10, 11, 29). We found that overall diversity of the supragingival fungal plaque communities was relatively low when compared to bacterial plaque communities (32). On average, four species were found to comprise each sample, with the predominant taxa across all samples being *C. albicans*. Similar to our findings, a previous study that examined the impacts of HIV/HAART on the oral mycobiome (oral rinse) found that generally only 1–3 species dominated the composition of each sample. This reduction in community composition was found to be significantly affected by multiple clinical factors, one being HAART treatment (29). Despite the low fungal diversity, our results exhibited significant differences in community composition of CA-PD samples when comparing HI and HEU children. There was a consistent reduction in species richness and evenness with disease (both HIV infection and caries). HI samples had the lowest diversity while HEU samples had the highest, with all three HIV categories exhibiting a reduction in diversity as caries progressed. Previous studies completed by us and others have identified this same pattern of reduced fungal diversity with caries progression (8, 9) and a reduction of both bacterial and fungal oral microbiome diversity with HIV infection (31, 33). Reduction of microbiome diversity with disease is not exclusive to the oral environment and has been observed in the gut, lungs, and skin (34 – 36). A reduction in microbial diversity can lead to changes in immune activation, increased inflammation response, and microbial capabilities that allow for survival and proliferation of opportunistic and pathogenic taxa that are capable of antagonizing and outcompeting beneficial flora. This can lead to a detrimental shift in community composition that promotes a diseased state (36, 37). Many of the clinical signs of HIV infection manifest in the oral cavity and have been shown to induce microbial dysbiosis resulting in loss of microbial diversity. An example of this is oral candidiasis, in which immunosuppression leads to a reduction of bacterial taxa which are capable of competing with and controlling the overgrowth of *C. albicans* (38). While the HI children in this study did not have oral candidiasis, our results offer insight into the probable dysbiotic shift occurring in the oral microbiome that may allow for conditions in which oral candidiasis can initiate and proliferate in HIV infection. There is also evidence to suggest that HIV infection adversely affects salivary gland function which can lead to conditions such as xerostomia (dry mouth) (39). Saliva is essential to maintaining tooth health due to its high buffering capacity, which helps to maintain a neutral pH within the tooth biofilm and reduce caries initiation (40). Saliva also helps to maintain a balanced oral microbiome by the removal of dietary carbohydrates and unattached bacteria and through delivery of proteins to the tooth surface which can prevent binding of pathogenic taxa and provide antimicrobial action. It is plausible to hypothesize that potential reduction of salivary gland function and reduced salivary flow driven by HIV infection can negatively impact the homeostasis of the oral microbiome and can act as a catalyst for dysbiosis. Our results suggest that mycobiome dysbiosis in HI samples is elevated in comparison to HEU and HUU samples as evidenced by reduced diversity. Further research to determine factors driving diversity reduction of the oral mycobiome in HI children will be useful in verifying the mechanism associated with increased caries risk.

*C. albicans* was the dominant species in the data, with the remainder of the community comprised of a varying diversity of lower frequency ASVs. The highest abundance of *C. albicans* was found in samples collected from children infected with HIV on HAART (HI children), followed by samples collected from children who were HUU, with the lowest abundance of *C. albicans* observed in samples from the HEU group. Among samples collected from HEU children, the most abundant ASV was *C. albicans* ASV2 as opposed to *C. albicans* ASV1 in samples from HI and HUU children. Though not significant, this shift in the dominant ASV between HEU and HI/HUU groups is intriguing and may serve as an excellent starting point to determine to what degree ASVs might differently affect the pathogenic potential of the biofilm, potentially having predictive value for health outcomes. Previous studies have found heterogeneity among genotypes of *C. albicans*, with associated differences in virulence capabilities, pH requirements, and colonization capabilities (41, 42). Another recent study examining bacteria of the gut microbiome for HEU and HUU children from infancy through 62 wk found that while there were no significant differences in alpha diversity between HUU and HEU groups, there were significant differences in the abundance of individual taxa (43). These differences were most divergent in early infancy and eventually converged over time. It has been previously documented that HEU children have increased immunological impairments, reduced accumulation of maternal IgG, and increased microbial translocation when compared to their HUU counterparts (23, 44), and it is plausible that these alterations could in turn stimulate environmental alterations contributing to shifts in *C. albicans* ASV distribution in HEU children and could potentially explain their reduced caries risk.

A total of 12 ASVs were identified as being significantly differentially abundant in HIV infection and exposure categories or in plaque categories. Of these 12 ASVs, only one ASV was significantly abundant between the three HIV study groups*—R. colostri*, which was significantly enriched in HEU and HI samples and was absent in HUU samples. *R. colostri* was also significantly abundant in relation to plaque category as it was only identified in health (CF-PF). *R. colostri,* formerly named *Rhodotorula colostri,* is a basidiomycete yeast originally isolated from human colostrum (45). This species has also been found as an endophytic yeast commonly living on the skin of fruits like apples and pears (46). It is interesting that this species was originally isolated from human milk colostrum, as 83% of the children in this study were breastfed. The presence of this species in both milk and on fruits suggests the possibility of acquisition through diet. Furthermore, the absence of *R. colostri* in HUU children suggests that HIV exposure may play a role in modulating its biofilm colonization and pervasiveness. In addition to *R. colostri,* two other ASVs were found to be significantly enriched in health, *A. penicillioides* ASV1 and *A. penicillioides* ASV2. This species is a filamentous fungus that has been previously associated with healthy non-diseased samples in oral and gut mycobiome studies (47, 48). Compellingly, this species has exhibited antibacterial activity against gram-positive and gram-negative human pathogens highlighting its potential as a biomedically relevant and health-promoting fungus and could warrant further study as a possible oral probiotic candidate (49).

An additional four ASVs were significantly enriched in mouths with dentin lesions. Two of these ASVs belonged to *C. albicans* (ASV16 and ASV34) and were present in highest abundance in HUU and HI samples. *N. desmazieri* ASV1 and *C. exasperatum* ASV1 showed highest abundance in CA-PD samples followed by CF-PF samples and were found in highest abundance in HEU samples. *N. desmazieri* (basionym: *Ascochyta desmazieri*) are characterized by their production of one-septate conidia. This species has not been well-studied in relation to humans but has been isolated from human nails and the respiratory tract (50). In a previous study of ours, *C. exasperatum* was identified as one of the most abundant fungal taxa in supragingival plaque samples isolated from US children but was not significantly associated with any stage of dental caries (8). This species has recently been identified in samples from oral squamous cell carcinomas (47). More research is needed to determine the significance of this species in oral health. The remaining four significantly abundant taxa were all *C. albicans* ASVs and were not strongly correlated with tooth health or disease.

While only 12 ASVs showed statistical significance for differential abundance, we found strong associations for some non-significant ASVs. For example, the highest number of ASVs associated with tooth health (CF-PF, CAE-PF, and CA-PF) were found in HEU samples. Surprisingly, HUU samples had the highest number of ASVs associated with progressed caries (CA-PE and CA-PD). HI samples were intermediate, having ASVs found in both tooth health and disease. The taxonomic differentiation of HEU communities from HI/HUU communities, their higher diversity, and higher number of health-associated species show these communities to be distinct from those associated with HUU and HI children and suggest a possible health advantage. Interestingly, for the children studied here, this distinctiveness for HEU communities aligns with a lower incidence of caries for primary teeth at age six or less (27). Specifically, prevalence was lowest for HEU followed by HUU and then HI. Numerous studies have shown a broad range of elevated HIV-1-specific T cell responses for HEU children when compared to HUU children (24). For example, HEU children show a higher antibody response to certain vaccines when compared to their HUU counterparts (24, 51). Consequently, it is possible that immunological alterations for HEU children primed by HIV exposure *in utero* or at birth could alter or hinder plaque biofilm formation, which could in turn reduce caries experience. Of note, Clerici et al. (52) showed that the breadth and magnitude of T cell responses appear to be greatest soon after birth and absent in children with an average age of 7.2 years, which aligns with our observation of lower caries incidence for primary teeth in children aged six or less (52).

There were two limitations to this study. The first was low sample size, which was due to unmeasurable concentration of fungal ITS1 PCR product after amplification for the majority (85.7%) of plaque samples collected (888 samples were evaluated). This low concentration was not observed in a previous study of ours focusing on USA children where the majority of samples produced measurable PCR product (8) and warrants further investigation. The second limitation stems from a limited knowledge base for oral fungi. For example, limited sequence data are available, and a curated database for oral fungi does not exist. Hopefully, this limitation can be addressed as more fungal genomes are sequenced. Future studies that examine both immune system response and taxonomic and gene expression changes in the fungal and bacterial plaque microbiomes with age, duration of breastfeeding, HAART treatment, and high maternal viral load will help to elucidate how HIV infection and exposure contribute to caries initiation and progression risk.

## MATERIALS AND METHODS

### Sample collection and processing

The samples used in this study are part of the dental caries and its association with the oral microbiomes and HIV in young children in Nigeria (DOMHaIN) cohort (53). All samples used here were collected during the baseline visit (Visit 1). Detailed descriptions of the recruitment, training, sampling process, and plaque category determination were described previously (53). Briefly, plaque categories were determined based on the International Caries Detection and Assessment System (ICDAS) and decayed, missing, and filled teeth (DMFT) scores. PF is a plaque sample collected from a tooth surface that is caries-free (ICDAS = 0). PE is a plaque sample collected from a tooth surface with an active enamel lesion (ICDAS = 1–3). PD is a plaque sample collected from a tooth surface with an active dentin lesion (ICDAS = ≥4). Secondly, children were classified by their caries status. A child without caries activity is classified as caries-free (CF; DMFT = 0). A child with enamel lesions only is classified as caries-active in enamel (CAE; DT = 0, MFT ≥ 0). A child with at least two active and unrestored dentin lesions is classified as caries-active in dentin (CA; DT ≥ 2, MFT ≥ 0). Each plaque sample is then able to be placed into one of six categories: caries-free child: plaque sample collected from a caries-free tooth surface (CF-PF); caries-active in enamel: plaque sample collected from a caries-free tooth (CAE-PF); caries-active in enamel: plaque sample collected from an active enamel lesion (CAE-PE); caries-active in dentin: plaque sample collected from a caries-free tooth (CA-PF); caries-active in dentin: plaque sample collected from an active enamel lesion (CA-PE); and caries-active in dentin: plaque sample collected from an active dentin lesion (CA-PD). Children exposed to antifungals up to 3 months prior to sampling were excluded from the study.

### DNA isolation and library preparation

Genomic DNA was isolated according to manufacturer’s protocol using the Qiagen DNeasy PowerBiofilm Kit (Qiagen, USA). Extraction blanks were included for each kit to ensure the absence of external contamination. Single-step PCR reactions were used to amplify the fungal ITS1 region using the following primer pair: ITS1F 5′-CTTGGTCATTTAGAGGAAGTAA-3′ and ITS2 5′-GCTGCGTTCTTCATCGATGC-3′ (11). The primers were designed to include custom indexes and Illumina adapters as outlined previously (54). A positive control of pure isolated *C. albicans* genomic DNA and a negative control of ultra-pure water were run with each set of PCR reactions to ensure that the PCR protocol worked appropriately and that there was no external contamination. After PCR amplification, the concentration of the reaction product was measured using Qubit 3.0 (ThermoFisher, USA). Samples that yielded a measurable concentration were run on a 2% TAE agarose gel. Qiagen’s QIAquick Gel Extraction Kits (Qiagen, USA) were used to extract target bands according to the manufacturer protocol. Samples were prepared for sequencing following Illumina’s NextSeq and MiSeq Denature and Dilute Libraries Guides. Read 1, Read 2, and Index custom primers were spiked into the sequencing reagent cartridge as outlined in Kozich et al. (54).

### Sequencing, sequence analysis, and taxonomic identification

Amplicon sequencing was completed on the Illumina MiSeq platform (Illumina, San Diego, CA) using a V2 500-cycle kit (250 base pair, paired-end reads). Demultiplexed reads were imported into QIIME2 (version: 2021.4) (55) for further sequence processing. Sequence denoising and quality control were performed using the Divisive Amplicon Denoising Algorithm (DADA2) pipeline in QIIME2 (56). DADA2 and QIIME2 were used to merge and trim reads, remove chimeras, cluster reads into ASVs, generate representative sequences for each ASV, and generate a read count table (55, 56). Taxonomic assignment of ASVs was completed using the dynamic UNITE fungal database QIIME2 release (February 2020 release) (57). The database was used to fit and train a Naïve-Bayes taxonomic classifier.

### Statistical analysis

Before completing diversity analyses for the samples, they were rarefied to an even sampling depth. The minimum read depth across all samples was 3,901. Alpha rarefaction curves were generated at this depth to ensure full diversity was captured. Statistical analysis of the reads was completed using QIIME2 and R Studio (version 2021.09.2) (58). Alpha diversity metrics were calculated in QIIME2 using the Shannon index and observed species metrics (59). Statistical significance of alpha diversity was calculated using the non-parametric, pairwise, Kruskal–Wallis test (60). Alpha diversity plots were generated using the Phyloseq (version: 1.38.0) and ggplots2 (version: 3.3.5) packages in R (version: 4.1.2 [2021–11-01]) (61 – 63). Beta diversity metrics were calculated in QIIME2 using the Bray–Curtis dissimilarity (64). Statistical significance was tested using pairwise PERMANOVAs. Neighbor joining dendrograms were constructed using associated variance components. PCoA plots were generated using Bray–Curtis dissimilarity in the phyloseq and ggplots2 packages in R. Hierarchical cluster analysis was performed using Bray–Curtis dissimilarity using the MicrobiotaProcess package (version 1.6.5) in R (65).

Differential abundance testing was performed using the DESeq2 package (version: 1.34.0) in R (66). *P*-values were generated using 10,000 permutations. The data used for DESeq2 analysis was the unfiltered and unrarefied read table generated in QIIME2 after DADA2. The data were log-transformed using a variance stabilized transformation (VSD). All *P*-values were corrected for multiple comparisons using the False Discovery Rate (FDR) (67). Species relative abundance plots were made from the rarefied read count table. All singletons, doubletons, and ASVs with a relative abundance below 0.01% were removed from the table prior to generating plots. The top 64 most abundant ASVs were plotted in Excel.

## Data Availability

The data sets generated and analyzed in the current study are available in the Sequence Read Archive (SRA) at NCBI under accession number PRJNA955275.

## SUPPLEMENTAL MATERIAL

Table S1 spectrum.01491-23-s0001.xlsx Plaque category and child clinical data associated with samples.

Table S2 spectrum.01491-23-s0002.xlsx Rarefied ASV counts.

Table S3 spectrum.01491-23-s0003.xlsx Differential taxon abundance tests.

ASM does not own the copyrights to Supplemental Material that may be linked to, or accessed through, an article. The authors have granted ASM a non-exclusive, world-wide license to publish the Supplemental Material files. Please contact the corresponding author directly for reuse.
